# Power and sample size calculation for incremental net benefit in cost effectiveness analyses with applications to trials conducted by the Canadian Cancer Trials Group

**DOI:** 10.1186/s12874-023-01956-y

**Published:** 2023-08-03

**Authors:** Louis Everest, Bingshu E. Chen, Annette E. Hay, Matthew C. Cheung, Kelvin K. W. Chan

**Affiliations:** 1 Odette Cancer Centre, Sunnybrook Health Sciences Centre, 2075 Bayview Ave, Toronto, ON M4N 3M5 Canada; 2 Department of Public Health Sciences, Canadian Cancer Trials Group, Queen’s, University, Kingston, ON Canada; 3 University of Toronto, Toronto, ON Canada; 4 Canadian Centre for Applied Research in Cancer Control, Toronto, ON Canada; 5 Cancer Care Ontario, Toronto, ON Canada

## Abstract

**Background:**

Historically, a priori power and sample size calculations have not been routinely performed cost-effectiveness analyses (CEA), partly because the absence of published cost and effectiveness correlation and variance data, which are essential for power and sample size calculations. Importantly, the *empirical* correlation between cost and effectiveness has not been examined with respect to the estimation of value-for-money in clinical literature. Therefore, it is not well established if cost-effectiveness studies embedded within randomized-controlled-trials (RCTs) are under- or over-powered to detect changes in value-for-money. However, recently guidelines (such as those from ISPOR) and funding agencies have suggested sample size and power calculations should be considered in CEAs embedded in clinical trials.

**Methods:**

We examined all RCTs conducted by the Canadian Cancer Trials Group with an embedded cost-effectiveness analysis. Variance and correlation of effectiveness and costs were derived from original-trial data. The incremental net benefit method was used to calculate the power of the cost-effectiveness analysis, with exploration of alternative correlation and willingness-to-pay values.

**Results:**

We identified four trials for inclusion. We observed that a hypothetical scenario of correlation coefficient of zero between cost and effectiveness led to a conservative estimate of sample size. The cost-effectiveness analysis was under-powered to detect changes in value-for-money in two trials, at willingness-to-pay of $100,000. Based on our observations, we present six considerations for future economic evaluations, and an online program to help analysts include a priori sample size and power calculations in future clinical trials.

**Conclusion:**

The correlation between cost and effectiveness had a potentially meaningful impact on the power and variance of value-for-money estimates in the examined cost-effectiveness analyses. Therefore, the six considerations and online program, may facilitate a priori power calculations in embedded cost-effectiveness analyses in future clinical trials.

**Supplementary Information:**

The online version contains supplementary material available at 10.1186/s12874-023-01956-y.

## Highlights

• Analysts may use the online program presented in the present study to examine the a priori power of cost-effectiveness analyses.

• Analysts may potentially apply the considerations presented in this paper in the planning stage of future cost-effectiveness analyses.

**Supplementary Information:**

The online version contains supplementary material available at 10.1186/s12874-023-01956-y.

## Background

The increasing cost of anticancer agents over the past two decades has generated discussion in the literature regarding the value of the novel anticancer therapies [1]. Specifically, concerns in literature have been presented due to the disproportionally modest survival benefits of novel cancer therapeutic agents, compared to the substantial increases in cost [2]. However, phase III anticancer trials are conventionally designed to detect improvements in efficacy and not necessarily changes in value-for-money [3, 4].

Currently, CEAs embedded in cancer trials are commonly completed without formal sample size and power calculations [5]. However, a recent ISPOR Good Research Practices Task Force report has suggested the inclusion of sample size calculations for CEAs embedded in clinical trials [5]. Additionally, research grant funding agencies may commonly request statistical sample size justifications even for secondary economic evaluation endpoints in cancer clinical trials [personal communication: Matthew Cheung, Co-chair of Committee on Economic Analysis, Canadian Cancer Trials Group, Nov 11, 2022]. However, there is currently a paucity of information in published literature with respect to empirical estimates of sample size parameters [5, 6]. Therefore, analysts may not be able to examine the power of CEAs embedded in clinical trials, despite its recognised importance in literature [5]. Additionally, the likelihood a new treatment is cost effective based on Bayesian methods, may be challenging to estimate because prior variance and covariance distributions are also not well established in literature [6, 7].

Importantly, because information regarding variances of cost and co-variances of cost and effectiveness are often not well established in the literature, a priori sample size or power calculations for cost-effectiveness analysis endpoints typically are not done or only partly done by making some assumptions regarding cost differences between the experimental and control groups and the corresponding variances [6, 8]. Additionally, because most new cancer treatments improve survival and increase costs compared to the standard, there is reasonable theoretical justification that covariance may be non-ignorable in these trials [9]. Therefore, in the absence of a priori power calculations, cost-effectiveness analyses may be under- or over-powered to detect changes in value [9]. Additionally, because many new cancer agents enter the market with incremental cost effectiveness ratios near willingness-to-pay thresholds, cost-negotiations resulting in lower drug prices may reduce the associated power of cost-effectiveness analyses [10].

An understanding of the relationship between the variances of and correlation between incremental cost and incremental effectiveness and their influence on power and sample size is not currently available for cancer trials with embedded economic evaluations [11]. To our knowledge, the Canadian Cancer Trials Group (CCTG) is currently the only oncology trial group with a standing committee, the Committee on Economic Analysis, dedicated to designing cost-effectiveness analyses to be embedded within randomized controlled trials with collection of cost and resource utilization prospectively. Therefore, we aimed to examine power calculations in cost-effectiveness analyses embedded within RCTs conducted by the CCTG, in order to better understand and facilitate future health technology assessments.

In this paper, we demonstrate the calculation of power and sample size of cost-effectiveness analyses based on the paradigm of incremental net benefit developed by Willan and Lin [11] using original trial cost and effectiveness individual patient data from phase III trials conducted by the CCTG [12–19]. The statistical model is summarized in the Methods section, followed by an application to cost-effectiveness analyses conducted by CCTG with respect to correlation coefficients and willingness-to-pay values in the Results section. We then discuss the practical implications of our demonstration in the Discussion section, providing guidance on the design of trial-based economic analyses and an online resource to enable sample size and power calculations.

## Methods

### Selection of Studies and Parameter Calculation

The present study examined all RCTs conducted by CCTG with an embedded cost-effectiveness analysis. Variance and correlation of effectiveness and costs were derived from original-trial data. The primary analysis examined the power of the cost-effectiveness analysis based on the incremental net benefit method, with exploration of alternative correlation and willingness-to-pay values.

### Statistical Methods of Calculating the Sample Size and Power based on the Paradigm of Incremental Net Benefit

Conventionally, incremental cost-effectiveness ratios (ICERs), and associated confidence intervals, are a common method of quantifying value and uncertainty respectively, in cost-effectiveness analyses of anticancer agents [4, 6]. In a two-arm randomized controlled trial, let the mean effectiveness of the treatment and standard arms be ${E}_{1}$ and ${E}_{0}$ respectively. Additionally, the mean in costs in the treatment and standard arms are represented as ${C}_{1}$ and ${C}_{0}$ respectively. The ratio between the change in mean cost and the change in mean effectiveness is the ICER, defined as:

$$ICER=\frac{{C}_{1}-{C}_{0}}{{E}_{1}-{E}_{0}}=\frac{\Delta C}{\Delta E}$$

where $\Delta C={C}_{1}-{C}_{0}$ and $\Delta E={E}_{1}-{E}_{0}$ are the cost and effectiveness difference between the treatment and the control groups, respectively.

The present analysis quantifies cost-effectiveness through the incremental net benefit (INB) method. This method was selected because sample size calculations based on the INB method and the willingness-to-pay value (or threshold), are well established in statistical literature [4], and are one of the commonly used methods, when sample size calculations are conducted in cost-effectiveness analyses [20]. In cost effectiveness analyses, the willingness-to-pay value is typically defined as the amount of money that a decision maker or society is willing to pay for a 1-unit improvement in efficacy [21, 22]. For a two-arm randomized controlled trial, the INB is defined as

1 $$b\left(\lambda \right)=\lambda \cdot \left(\Delta E\right)-\left(\Delta C\right)$$

Importantly, the ICER may be calculated as a special case of the INB. Specifically, the ICER may be calculated as the horizontal intercept of the plot of b(λ) (y axis) and λ (x axis).Therefore, when the INB is calculated as $\lambda \cdot \left(\Delta E\right)-\left(\Delta C\right)=0$, willingness-to-pay value λ is equivalent to the ICER. Furthermore, the confidence limits for $b\left(ICER\right)$ cross the horizontal axis at the Fieller limits for a specified ICER, allowing for further inferences [6, 11, 23]. Therefore, without the loss of generality, the INB is used in the present analysis; however, the practical problems encountered when implementing the ICER method are similarly applicable [6, 11]. However, because ICER is a ratio of the cost and effectiveness, statistical inferences such as the corresponding standard error may be difficult to obtain. [6, 11]. Additionally, standard errors may be unreasonable in some cases (e.g., when the change in efficacy is close to zero, resulting in a standard error that is close to infinity).

The hypothesis test of the cost-effectiveness for a willingness-to-pay value may be examined as:

$$\begin{array}{c}{H}_{0}:b\left(\lambda \right)\le 0,versus\hspace{0.17em}{H}_{1}:b\left(\lambda \right)>0\end{array}$$

where the alternative hypothesis may be interpreted as suggesting the treatment is demonstrated to be cost-effective. Additionally, failing to reject the null hypothesis implies that the cost-effectiveness of the treatment, when compared to the control, is at or below the willingness-to-pay threshold, barring the lack of statistical power to reject the null hypothesis. Further, in the present study, the hypothesis test ${H}_{0}:b\left(\lambda \right)\ge 0,versus\hspace{0.17em}{H}_{1}:b\left(\lambda \right)<0$ is also examined in the CO.17 and CO.17 KRAS trials. This hypothesis test was included to examine the impact of the correlation coefficient on non-cost-effective trials. Importantly, the latter hypothesis test is unrealistic in CEAs, however the trends are generalizable to the former hypothesis test with respect to the impact of the correlation coefficient.

### Variance and Sample Size Calculations

For a study with sample size $n$, where ${n}_{0}$ and ${n}_{1}$ represent the sample sizes of the control and treatments groups respectively, the variance of the INB $b\left(\lambda \right)$ can be estimated as:

2 $$\sigma_{b\left(\lambda\right)}^2=\frac1{n_0}\left(\lambda^2\sigma_{E_0}^2+\sigma_{C_0}^2-2\lambda\rho_0\sigma_{E_0}\sigma_{C_0}\right)+\frac1{n_1}\left(\lambda^2\sigma_{E_1}^2+\sigma_{C_1}^2-2\lambda\rho_1\sigma_{E_1}\sigma_{C_1}\right)$$

where ${\sigma }_{{E}_{j}}^{2}$ and ${\sigma }_{{C}_{j}}^{2},j=0,1$ are the variance of effectiveness and costs in the control and experimental groups, respectively. Additionally, ${\rho }_{j}$ is the correlation coefficient between effectiveness and cost in the group $j,j=0,\hspace{0.25em}1$. Conventionally, the variance of ICER is calculated through bootstrap methods or Fieller’s theorem in some cases. A derivation of the Fieller’s confidence limits when $\widehat{b}\left(\lambda \right)$ crosses the horizontal axis is presented in Willan (2006) [24] as well as Zethraeus and Löthgren [23]. Variance and correlation of effectiveness and costs were derived from original-trial data using intent-to-treat analyses, over the entire trial time horizon. Detailed derivations and formulas of these parameters are presented by Willan [9]. Additionally, details with respect to the parameter estimates are presented in the associated CEAs of the examined trials [16–19]. The smallest important difference in incremental net benefit is defined as ${b\left(\lambda \right)}_{\delta }=\lambda \times \left(\mathrm{\Delta E}\right)-\left(\mathrm{\Delta C}\right)$. Therefore, as examined by Willan and Lin (2001), to test the one-sided hypotheses at the $\alpha$ level and $\left(1-\beta \right)\times 100\mathrm{\%}$ power, the total sample size is given by:

3 $$n=\frac{{\left({z}_{1-\alpha }+{z}_{1-\beta }\right)}^{2}\cdot {\sigma }_{b\left(\lambda \right)}^{2}}{b{\left(\lambda \right)}_{\delta }^{2}}$$

where the type I error, $\alpha$ in Eq. (3), is the probability of claiming that the treatment cost-effective if the null hypothesis is true, which is usually set to be $\alpha =0.05$. The type II error, $\beta$ in Eq. (3), is the probability of failing to reject the null hypothesis, when the true INB is equal to or less than ${b\left(\lambda \right)}_{\delta }$. Therefore, ${z}_{1-\alpha }$ and ${z}_{1-\beta }$ are quantiles of a standard normal distribution with respect to $\left(1-\alpha \right)$ and the power, respectively.

In general, a priori assumptions based on existing literature regarding $\Delta E$ and variance of $\Delta E$, are used to inform the primary endpoint of the efficacy sample size calculations of the clinical trial. Specifically, in phase III clinical trials, efficacy sample size calculations are typically based on the target hazard ratio and the assumption of an exponential survival distribution [3]. Therefore, in the present analysis, the sample size calculation is based on the assumption that survival time follows an exponential distribution. In comparison, the treatment effectiveness $\Delta E$ and variance ${\sigma }_{\Delta E}^{2}$ were calculated analytically, based on individual patient data.

To have a better understanding on what roles the correlation coefficients will play in the sample size and power determination, we make some simplification based on the following assumptions:

1. The study will be balanced between the control and experimental arms ${n}_{0}={n}_{1}=\frac{n}{2}$.
2. The costs in the control and experimental arms have the same variance ${\sigma }_{{C}_{0}}^{2}={\sigma }_{{C}_{1}}^{2}={\sigma }_{C}^{2}$
3. The effectiveness in the control and experimental arms have the same variance ${\sigma }_{{E}_{0}}^{2}={\sigma }_{{E}_{1}}^{2}={\sigma }_{E}^{2}$
4. The correlation coefficients in the control and experimental arms are the same: ${\rho }_{0}={\rho }_{1}=\rho$

These assumptions have been made to facilitate illustrative comparisons with respect to the impact of the correlation coefficient on sample size calculation and should be applied to external cost-effectives analyses cautiously. Based on these assumptions, we can re-write variance formula Eq. 2 as

4 $${\sigma }_{b\left(\lambda \right)}^{2}=\frac{4}{n}\left({\lambda }^{2}{\sigma }_{E}^{2}+{\sigma }_{C}^{2}-2\lambda \rho {\sigma }_{E}{\sigma }_{C}\right)$$

Since $b{\left(\lambda \right)}_{\delta }=\lambda \cdot \left(\Delta E\right)-\left(\Delta C\right)$, let $z={\left({z}_{1-\alpha }+{z}_{1-\beta }\right)}^{2},$ for example, when one-sided alpha = 0.05 and power = 80%, we have z = 6.18 and $4\times z$ = 24.72. Therefore, we can replace the quantities in the sample size Eq. 3 with the corresponding terms for cost differences and the effectiveness differences in incremental net benefit analysis and obtain the following sample size formula

5 $$n=4\cdot z\cdot \frac{{\lambda }^{2}\pi {\sigma }_{E}^{2}+{\sigma }_{C}^{2}-2\lambda \rho \sqrt{\pi }{\sigma }_{E}{\sigma }_{C}}{{\lambda }^{2}{\left(\Delta E\right)}^{2}+{\left(\Delta C\right)}^{2}-2\lambda \left(\Delta E\right)\left(\Delta C\right)}$$

where $\pi =\frac{{n}_{e}}{{d}_{e}}$, and denotes the ratio of the total expected sample size $\left({n}_{e}\right)$ and the expected number of events $\left({d}_{e}\right)$. The parameter $\pi$ will be fixed once the design for the primary effectiveness endpoint is finalized. For example, in a trial with sample size ${n}_{e}=500$ and the final analysis will be triggered when ${d}_{e}=400$ events are observed, then $\pi =\frac{500}{400}=1.25$. Furthermore, as examined by Willan & Lin [11], the corresponding power function is given by:

6 $$P\left(\delta \right)=\Phi \left(\frac{\widehat{b}{\left(\lambda \right)}_{\delta }}{\sqrt{V[\widehat{b}\left(\lambda \right)]}}-{Z}_{1-\alpha }\right)=\Phi \left(\frac{\lambda \cdot \left(\Delta E\right)-\left(\Delta C\right)}{\sqrt{\frac{4}{n}\times \left({\lambda }^{2}{\sigma }_{E}^{2}\hspace{0.25em}+{\sigma }_{C}^{2}-2\lambda \rho {\sigma }_{E}{\sigma }_{C}\right)}}-{Z}_{1-\alpha }\right)$$

where $\Phi \left(\cdot \right)$ is defined as the cumulative distribution function for a standard normal random variable. The power curve gives the probability of rejecting the hypothesis ${H}_{0}:\hspace{0.25em}b\left(\lambda \right)\le 0$ in favour of the hypothesis ${H}_{1}:\hspace{0.25em}b\left(\lambda \right)>0$, at the level $\alpha$, for a given smallest important difference in incremental net benefit value, $b{\left(\lambda \right)}_{\delta }$ [11, 25]. Therefore, in the present study, the smallest important difference may be conservatively calculated as a function of the observed $\Delta E$ and $\Delta C$ values, because discussion in literature exists regarding the best method of defining $b{\left(\lambda \right)}_{\delta }$ [24]. However, because the intent of the present analysis was to examine the practical implications of the correlation coefficient between effectiveness and cost, with respect to a priori sample size or power calculations for cost-effectiveness analysis, the results may be generalizable to other methods of defining $b{\left(\lambda \right)}_{\delta }$. Additionally, as a sensitivity analysis the present study also examined a frequentist method of $b{\left(\lambda \right)}_{\delta }.$ Briefly, this frequentist method is characterized as the minimum $b{\left(\lambda \right)}_{\delta }$ value that satisfies Eq. 6 [6]. Further information on this frequentist method of $b{\left(\lambda \right)}_{\delta }$ is available in Lachin [26].

Additionally, the methods examined in the present study may be extended to non-censored data based on the mean parameter estimates, as examined in Willan [6, 11].

## Results 

### Summary of included trials

In the present analysis, we included all the trials for which CCTG had completed a cost-effectiveness analysis, including empirically calculated variances (of means of cost and effectiveness), correlation coefficients, as well as means of the $$\Delta C$$ and $$\Delta E$$. In total, we identified four trials, and one retrospective subgroup analysis from one of the four trials for inclusion. The present analysis found that the standard deviation for the cost of the experimental arm ranged from $10,000 to $35,000. The standard deviation for the effectiveness of the experimental arm was observed to range from 0.1-years to 2.72-years. The correlation coefficient between $$\Delta C$$ and $$\Delta E$$ were observed to be low to moderate in all the included trials (0.042 to 0.44) (Table 1).

**Table 1:** Summary of Cost and Survival differences for selected CCTG trials

Trial	n	Survival ${E}_{1}$ (Years)		Cost ${C}_{1}$ (CAD)		$\boldsymbol\rho$
		SE	$\boldsymbol\sigma$	SE	$\boldsymbol\sigma$	
BR.10	172	0.2930	2.717	3,757	34,840	0.124
BR.21	731	0.0325	0.622	558	10,668	0.203
LY.12^a^	519	0.0045	0.073	1,807	29,103	0.042
CO.17 (all patients)	567	0.0184	0.309	958	16,130	0.44
CO.17 (KRAS subgroup)	226	0.0296	0.316	1,598	16,987	0.43

*E*_1_ Experimental arm effectiveness, *C*_1_ experimental arm cost, $\rho$ Correlation Coefficient, *SE *Standard Error, $\sigma$ Standard deviation, *CAD* Canadian Dollars

^a^ LY.12 is a non-inferiority design. For LY.12, survival is defined as the restricted mean QALY from randomization to stem cell mobilization

### Correlation coefficient analysis

The correlation coefficient analysis of the present study examined value-for-money estimates changed, as a function of original-trial cost and effectiveness correlation. We applied the proposed variance formula for health economic to four different CCTG studies: BR.10, BR.21, CO.17 (all patients and a sub study of CO.17 KRAS wild type) and LY.12. First, we examined the impact of the correlation between the $\Delta C$ and $\Delta E$ on the variance of $b\left(\lambda \right)$. The results of the correlation coefficient analysis are summarized in Table 2. Additionally, contour plots examining the impact of $\rho$ and $b\left(\lambda \right)$ with respect to the variance of $b\left(\lambda \right)$ are presented in Fig. 4 of the Additional file 1: Appendix. Below we use the CO.17 trial (all patients) to demonstrate how the variance of $b\left(\lambda \right)$ changes as a function of the correlation coefficient $\rho$, which can vary from -1 (minimum) to 1 (maximum).

**Table 2:** Variance of the incremental net benefit estimates by correlation coefficient, assuming a willingness-to-pay threshold of $100,000

Trial	Variance		Relative Changes
	$\boldsymbol\rho\boldsymbol=\mathbf0$	$\boldsymbol\rho\boldsymbol=\boldsymbol o\boldsymbol b\boldsymbol s\boldsymbol e\boldsymbol r\boldsymbol v\boldsymbol e\boldsymbol d$	
BR.10	1,744,993	1,690,399	3%
BR.21	21,792	20,318	7%
LY.12	6,922	6,800	2%
CO.17 (all patients)	8,571	5,477	36%
CO.17 (KRAS subgroup)	22,780	14,610	36%

$$\rho$$ Correlation Coefficient

The trial CO.17 is a randomized controlled trial comparing the efficacy of cetuximab plus best supportive care (*n* = 287) versus best supportive care (*n* = 285) in patients with refractory advanced colorectal cancer. Assuming that the willingness-to-pay is $100,000 per life-year gained, then the variance of $$b\left(\lambda \right)$$ is given by

$$\begin{array}{c}{\sigma }_{b\left(\lambda \right)}^{2}=8,574-3,516\cdot \rho \end{array}$$

When $$\rho =0$$, the variance $${\sigma }_{b\left(\lambda \right)}^{2}=8,571$$. When $$\rho =0.44$$, the variance $${\sigma }_{b\left(\lambda \right)}^{2}=5,477$$, which is a decrease of 36.1%. When $$\rho =-1$$ or 1, the variance will be increased or decrease by 82.0%, respectively. Assuming that the willingness-to-pay is $200,000 per life-year gained, then the variance of $$b\left(\lambda \right)$$ is given by

$$\begin{array}{c}{\sigma }_{b\left(\lambda \right)}^{2}=28,778-7,032\cdot \rho \end{array}$$

Which is a linear function of the correlation coefficient $$\rho$$. When $$\rho =0$$, the variance is 28,778. When $$\rho =0.44$$, the estimated correlation coefficient from the trial data, the variance $${\sigma }_{b\left(\lambda \right)}^{2}=21,746$$, which is a decrease of 21.5%. When $$\rho =-1$$ or 1, the variance will be increased or decrease by 48.8%, respectively. The relative changes of the variance when $$\rho =0$$ and $$\rho =observed$$, at a willingness-to-pay value of $100,000, across all the examined trials is presented in Table 2. In the examined trials, the correlation coefficient accounted for a reduction range of 2% to 36% of the INB variance. Further, based on the CO.17 example, we appreciate that the larger the correlation coefficient, the greater the reduction of INB variance.

**Fig. 1:**
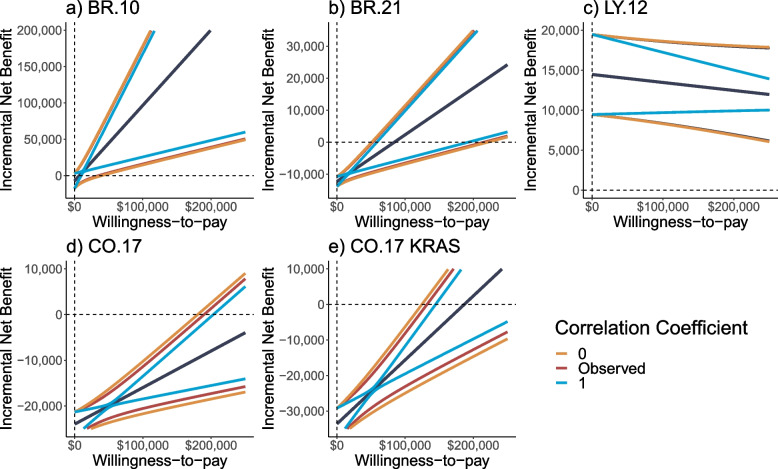
Impact of the correlation coefficient on the confidence intervals of the incremental net benefit across willingness-to-pay values

The relationship between the correlation coefficient and confidence intervals of the INB estimate, across a range of willingness-to-pay $$(\uplambda )$$ values ($0 to $250,000), are examined in Fig. 1. In all the included trials, when the willingness-to-pay increased from $100,000 to $200,000, the width of the confidence intervals of the INB estimate also increased. Additionally, in all the included trials when the correlation coefficient was assumed as $$\rho =0$$, the confidence intervals of the INB estimate, were slightly more conservative (i.e., wider), compared to the observed correlation coefficient value. Further, in all the included trials when the correlation coefficient was assumed as $$\rho =1$$, the confidence intervals of the INB estimate underestimated the observed confidence interval (i.e., too narrow). Inferences with respect to the ICERs of the examined trials are also possible, as the ICER and the Fieller limits are represented as the intersection at the horizontal axis of the INB estimate and confidence intervals, respectively [11, 23]. Additionally, the $$\Delta E$$ and $$\Delta C$$ values used to derive the INB estimates based on the cost-effectiveness analysis in all the examined trials, are presented in the (Additional file 1: Appendix Table 1).

### Sample size and power calculations

**Fig. 2:**
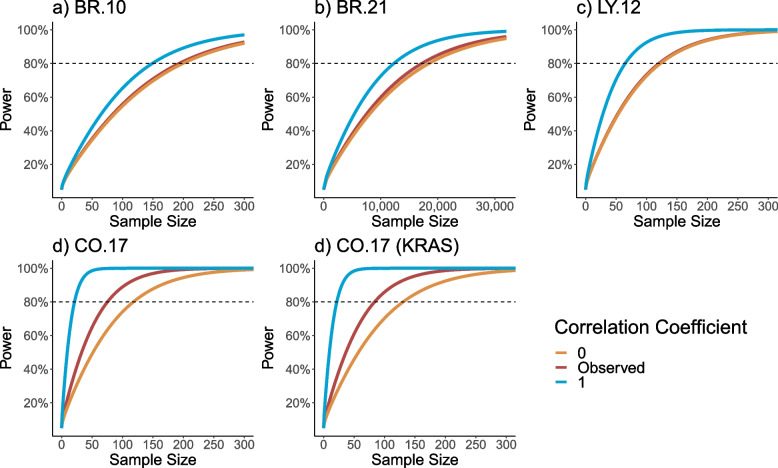
Impact of the correlation coefficient on the sample size power function curve, at the willingness-to-pay threshold of $100,000 Legend: Panels **a)**, **b)**, and **c)** were examined based on the hypothesis test: $${H}_{0}:\hspace{0.25em}b\left(\lambda \right)\le 0$$, versus $${H}_{1}:b\left(\lambda \right)>0$$. Panels **d)** and **e)** were examined based on the hypothesis test $${H}_{0}:\hspace{0.25em}b\left(\lambda \right)\ge 0$$, versus $${H}_{1}:b\left(\lambda \right)<0$$

In order to examine the relationship between the correlation coefficient and the power and sample size of the examined trials, we applied the power formula presented in Eq. 6, to the four CCTG trials (and one subgroup analysis), when $$\rho =0$$, $$\rho =observed$$, and $$\rho =1$$. This analysis examines the probability of rejecting the null hypothesis $${H}_{0}:b\left(\lambda \right)\le 0$$, versus $${H}_{1}:b\left(\lambda \right)>0$$ (and $${H}_{0}:b\left(\lambda \right)\ge 0,versus\hspace{0.17em}{H}_{1}:b\left(\lambda \right)<0$$ in the CO.17 and CO.17 KRAS trials), at willingness-to-pay threshold of $100,000 (Fig. 2).

In all the examined trials, when the correlation coefficient was assumed to equal the correlation coefficient observed in the associated cost-effectiveness analysis, the sample size needed to detect a change in the INB value decreased compared to $$\rho =0$$. In trials with correlation coefficients close to zero (LY.12: $$\rho =0.042$$) the sample size required to have 80% power to reject the null hypothesis, changed minimally. However, in trials with relatively higher correlation coefficients (CO.17 (all patients):$$\rho =0.44$$) the magnitude of the sample size reduction, in order to have 80% power to reject the null hypothesis, was also relatively higher. Additionally, this trend was also observed in all trials when the $$b{\left(\lambda \right)}_{\delta }$$ value was defined using a frequentist method, (Additional file 1: Appendix Fig. 1). This observation was expected because, based on formula 4, it is straightforward to appreciate the relationship between the sample size and the correlation coefficient $$\rho$$. As the correlation coefficient increases, the required sample size decreases. The relationships between the correlation coefficient and confidence intervals of the INB estimate, across a range of willingness-to-pay $$(\uplambda )$$ values ($0 to $250,000), are examined in Fig. 1.

**Fig. 3:**
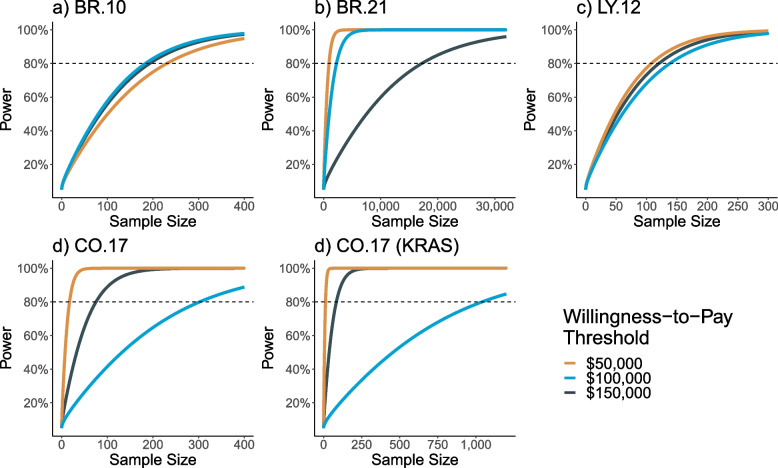
Impact of the willingness-to-pay threshold on the sample size power function curve, at the correlation coefficient observed in the respective trial Legend: Panels **a)**, **b)**, and **c)** were examined based on the hypothesis test: ${H}_{0}:\hspace{0.25em}b\left(\lambda \right)\le 0$, versus ${H}_{1}:b\left(\lambda \right)>0$. Panels **d)** and **e)** were examined based on the hypothesis test ${H}_{0}:\hspace{0.25em}b\left(\lambda \right)\ge 0$, versus ${H}_{1}:b\left(\lambda \right)<0$

In order to examine the relationship between the power and sample size of the included trials across willingness-to-pay thresholds, we also applied the power function presented in Eq. 6, to the four CCTG trials (and one subgroup analysis) at willingness-to-pay thresholds of $50,000, $100,000, and $150,000. Figure 3 examines the probability of rejecting the null hypothesis $${H}_{0}:\hspace{0.25em}b\left(\lambda \right)\le 0$$, versus $${H}_{1}:b\left(\lambda \right)>0$$ (and $${H}_{0}:b\left(\lambda \right)\ge 0,versus\hspace{0.17em}{H}_{1}:b\left(\lambda \right)<0$$ in the CO.17 and CO.17 KRAS trials), assuming the correlation coefficient observed in the cost-effectiveness analysis. The observed cost effectiveness analyses were under-powered (< 80%) to reject the null hypothesis at a willingness-to-pay value of $100,000 in two of the examined trials. Further, when the INB value was close to 0 at the examined willingness-to-pay threshold, as in BR.10 at a willingness-to-pay value of $100,000, the sample size needed to reject the null hypothesis increased considerably, compared to the other examined trials.

Furthermore, in order to examine the relationship between power and sample size of the examined trials, as a secondary analysis we also examined two-sided hypothesis testing in Additional file 1: Appendix Figs. 2 and 3. Specifically, the null and alternative hypothesis are modelled as $${H}_{0}:b\left(\lambda \right)=0$$, and $${H}_{1}:b\left(\lambda \right)\ne 0$$. In Fig. 2 the correlation coefficient was modelled as $$\rho =0$$, $$\rho =1$$, and $$\rho =\mathrm{observed}$$, and the willingness-to-pay threshold was examined as $100,000. In Fig. 3, the correlation coefficient was examined as $$\rho =observed$$ and the willingness-to-pay threshold was examined at $50,000, $100,000, and $150,000. In general, we observed similar trends to those examined in the primary analysis. Predictably, the sample sizes observed in the two-sided testing analysis were larger compared to the primary analysis.

## Discussion

In this paper we examine the application of the INB method for calculating sample size and power in economic evaluations, using original trial data from the CCTG. In general, the present analysis reported that the correlation coefficient between $\Delta E$ and $\Delta C$, had a potentially meaningful impact on the power and sample size calculations in the examined economic analyses. Specifically, when the correlation coefficient increased from $\rho =0$ to $\rho =observed$, the variance of the INB decreased. Additionally, when the correlation coefficient increased from $\rho =0$ to $\rho =observed$ the sample size needed to detect the smallest important differences in value also decreased. The present analysis examined the INB; however, by varying the willingness-to-pay value, inferences with respect to the ICER are also possible.

Because of the historical absence of a priori cost-effectiveness analysis power calculations in pivotal cancer trials [5, 11], we have also developed an easy-to-use online program (http://statapps.tk/icer_samplesize), for analysts to calculate power and sample size at the design stage of cost-effectiveness analyses. Additionally, as an illustrative example of the program, possible input parameters for the BR.21 trial is presented in Fig. 5 of the Additional file 1: Appendix. Importantly, the application of our proposed program may be informed by the empirical observations presented in the current study. As examined by Willan (2001), the proposed methods may be used to identify a subset of the trial population for cost-effectiveness analysis, in contrast to historical methods, which required larger sample sizes compared to the corresponding effectiveness analysis, to detect changes in cost-effectiveness [4, 11]. This program will also determine the standard deviation of the effectiveness outcome based on the design of the original clinical trial, including type I error rate, power, survival probability of the control arm at a given time point and the hazard ratio.

In the present analysis, we observed a low to modest correlation between $\Delta E$ and $\Delta C$ in all the examined trials. Based on the sample size analysis, assuming the correlation coefficient $\rho =0$ may be a conservative estimate to ensure sufficient power of cost effectiveness analyses. Additionally, when sufficient evidence of indication-specific correlation coefficients exists in literature, power calculations in cost-effectiveness analyses may consider correlation coefficients, in order to design more efficient economic evaluations when survival and costs are positively correlated. Further, as it is uncommon for cost and survival to be negatively correlated, negative correlation coefficients may only be applicable in unique clinical scenarios where a novel intervention would substantially reduce the utilization of a downstream expensive intervention while increasing survival. Assuming a negative correlation coefficient without sufficient biological justification will lead to an overly conservative large sample size, just as assuming a positive correlation coefficient without sufficient biological justification will lead to an insufficiently small sample size.

In the BR.21 trial at a willingness-to-pay threshold of $100,000, the sample size needed to reject the null hypothesis was considerably larger, compared to the sample size of the actual cost-effectiveness analysis. The interpretation of failing to reject the null hypothesis is that the cost-effectiveness of the treatment, when compared to the control, is not different from the willingness-to-pay threshold. Therefore, we cannot make a conclusion with respect to if a therapy is cost-effective at the examined willingness-to-pay threshold. Additionally, this sample size ballooning occurred when the INB estimate was near zero at the examined willingness-to-pay threshold. This observation may be relevant because novel first-in-class anticancer agents with no a priori pricing information conventionally enter the market with their prices based on the willingness-to-pay threshold [10]. In these scenarios, cost-effectiveness analysis may be under powered to reject the null hypothesis. Therefore, in the absence of a priori sample size or power calculations, cost-effectiveness analyses may not be able to identify if novel treatments are more or less cost-effective compared to the control treatments.

In two of the examined trials (and one subgroup population), the sample size required to reject the null hypothesis, was smaller compared to the corresponding efficacy analysis, at $$\rho =observed$$ and a willingness-to-pay threshold of $100,000 (i.e. the cost-effectiveness analyses were over-powered). In practice, it may not be feasible for analysts to embed cost-effectiveness analyses within clinical trials that require sample sizes larger than the corresponding efficacy analyses. However, a priori power calculations in cost-effectiveness analyses embedded within pivotal clinical trials may prioritize the development of cost-reduction therapies, and agents that preserve durable long-term response and survival. Additionally, when economic evaluations are over-powered, a priori power calculations may facilitate the identification of a population subset for sub-group cost-effectiveness analysis, in order to minimize wasting resources. Further, within a net benefit regression framework, power may also be derived based on a t-test or bootstrapping methods [27, 28]. The net benefit regression framework may also benefit similarly from the observations and proposed considerations presented in the current study.

Importantly, because the empirical parameter values observed in the present study could not be systematically compared to external studies, the validity of these estimates based on trial characteristics is not well established (e.g., the impact of sample size, dropout, or type of outcomes). This limitation highlights the need for future CEAs to report detailed cost and efficacy variance and covariance data, in order to iteratively refine a range of possible values and formal guidelines for CEA analysts. Additionally, changes in price during cost-negotiations may result in changes to the associated sample size and power estimates. Therefore, analysts may consider examining a range of possible parameter estimates.

The proposed a priori cost-effectiveness power and sample size formula utilizes the predicted variance of the cost, effectiveness, and the correlation between cost and effectiveness as well as the expected event rate. The present study addresses the gap in literature with respect to original trial data of cost and effectiveness variances, as well as the correlation between cost and effectiveness.

### Considerations

Based on the empirical exploration of original-trial data, we present six potential considerations for future individual patient-based economic evaluations embedded in clinical trials:

1. At the design stage of clinical trials, embedded CEAs may calculate $\Delta E$ and standard error of $\Delta E$ based on the efficacy assumptions for the primary efficacy endpoint statistical design.
2. At the design stage of clinical trials, embedded health care perspective CEAs may calculate $\Delta C$ based on the expected difference in the duration and costs of the experimental drug/regimen versus the control drug/regimen. If the price of the drug is not yet known at the time of the design, reviewers could identify a marketed drug from a comparable class (e.g., “me-too” drugs may base costs on the associated first-in-class drug) or a range of possible estimates, before adjusting for cost add-ons or offsets.
3. At the design stage of future comparable cancer CEAs embedded in clinical trials, standard error of the control and experimental costs could be based on the range of values observed in the trials from CCTG as reported in the present study ($500 to $4,000).
4. At the design stage of future comparable CEAs embedded in clinical trials, correlation coefficients between $\Delta C$ and $\Delta E$, could be based on the range of values we observed in the present study ($\rho =0$ to 0.5), where $\rho =0$ will lead to a conservative estimate of sample size when the true $\rho >0$. These estimates may be examined as a range of possible values and should be iteratively refined as additional estimates become available in literature.
5. When the sample size of an examined trial is defined based on the efficacy analysis, the corresponding a priori cost-effectiveness analysis power calculations and potential trial population subset may be completed using the INB approach such as using our online calculator application.
6. Future cost-effectiveness analysis based on individual patient data should consistently report their observed variances and correlation coefficients of $\Delta E$ and $\Delta C$ in order to provide additional information to facilitate future power calculations in future studies in related cancer settings.

## Conclusion

Based on our empirical observations of original-trial data, we present six potential considerations for future economic evaluations of clinical trials. Additionally, the online program presented in the paper may facilitate a priori calculations of power and sample size in future cost-effectiveness analyses.

### Supplementary Information

**Additional file 1.**

## Data Availability

The data that support the findings of this study are available from CCTG but restrictions apply to the availability of these data, which were used under license for the current study, and so are not publicly available. Data are however available upon reasonable request to the corresponding author (kelvin.chan@sunnybrook.ca) and with permission of CCTG as outlined in CTG-POL-0043 Data Sharing and Access Policy.
