# Supplementary material for: Power and sample size calculation for incremental net benefit in cost effectiveness analyses with applications to trials conducted by the Canadian Cancer Trials Group
Source: BMC Med Res Methodol. 2023 Aug 3;23:179. doi: 10.1186/s12874-023-01956-y (PMC10398980; doi:10.1186/s12874-023-01956-y)
Supplement: Supplementary file 1 — Additional file 1. [file 12874_2023_1956_MOESM1_ESM.docx]

**Power and sample size calculation for incremental net benefit in cost effectiveness analyses with applications to trials conducted by the Canadian Cancer Trials Group**

Appendix

Table 1: Incremental net benefit values as a function of the willingness-to-pay threshold

| Trial | ∆E (Years) | ∆C (CAD) | *b(λ*) Value | | |
| --- | --- | --- | --- | --- | --- |
|  |  |  | b(50,000) | b(100,000) | b(150,000) |
| BR.10 | 1.04 | 7,441 | 44,559 | 96,559 | 148,559 |
| BR.21 | 0.146 | 12,289 | -4,989 | 2,311 | 9,611 |
| CO.17 (all patients) | 0.08 | 23,969 | -19,969 | -15,969 | -11,969 |
| CO.17 (KRAS) | 0.18 | 33,617 | -24,617 | -15,617 | -6,617 |
| LY.12* | -0.01 | -14,464 | 13,964 | 13,464 | 12,964 |

∆E: effectiveness difference between the treatment and the control groups in years

∆C: cost difference between the treatment and the control groups in Canadian Dollars

*λ*: Willingness-to-pay threshold in dollars

*b( ):* Incremental net benefit function

* LY.12 is a non-inferiority design. For LY.12, survival is defined as the restricted mean QALY from randomization to stem cell mobilization.

Figure 1: Impact of the correlation coefficient on the sample size power function curve, at the willingness-to-pay threshold of $100,000, where $H_{0}: b\left( \lambda\right)=0, \mathrm{versus} H_{1}:b\left( \lambda\right)\neq0$, where ${b(\lambda)}_{\delta}$ was defined using a frequentist method.

Figure 2: Impact of the correlation coefficient on the sample size power function curve, at the willingness-to-pay threshold of $100,000, where $H_{0}: b\left( \lambda\right)=0$, versus $H_{1}:b\left( \lambda\right)\neq0$

Figure 3: Impact of the willingness-to-pay threshold on the sample size power function curve, at the correlation coefficient observed in the respective trial, where $H_{0}: b\left( \lambda\right)=0$, versus $H_{1}:b\left( \lambda\right)\neq0$

Figure 4: Contour plots of the correlation coefficient ($\rho$) and the willingness-to-pay value ($b\left( \lambda\right)$ ) with respect to the variance of $b\left( \lambda\right)$.

Figure 5: Illustrative example of possible input parameters to the online program (<http://statapps.tk/icer_samplesize>) for the BR.21 trial.


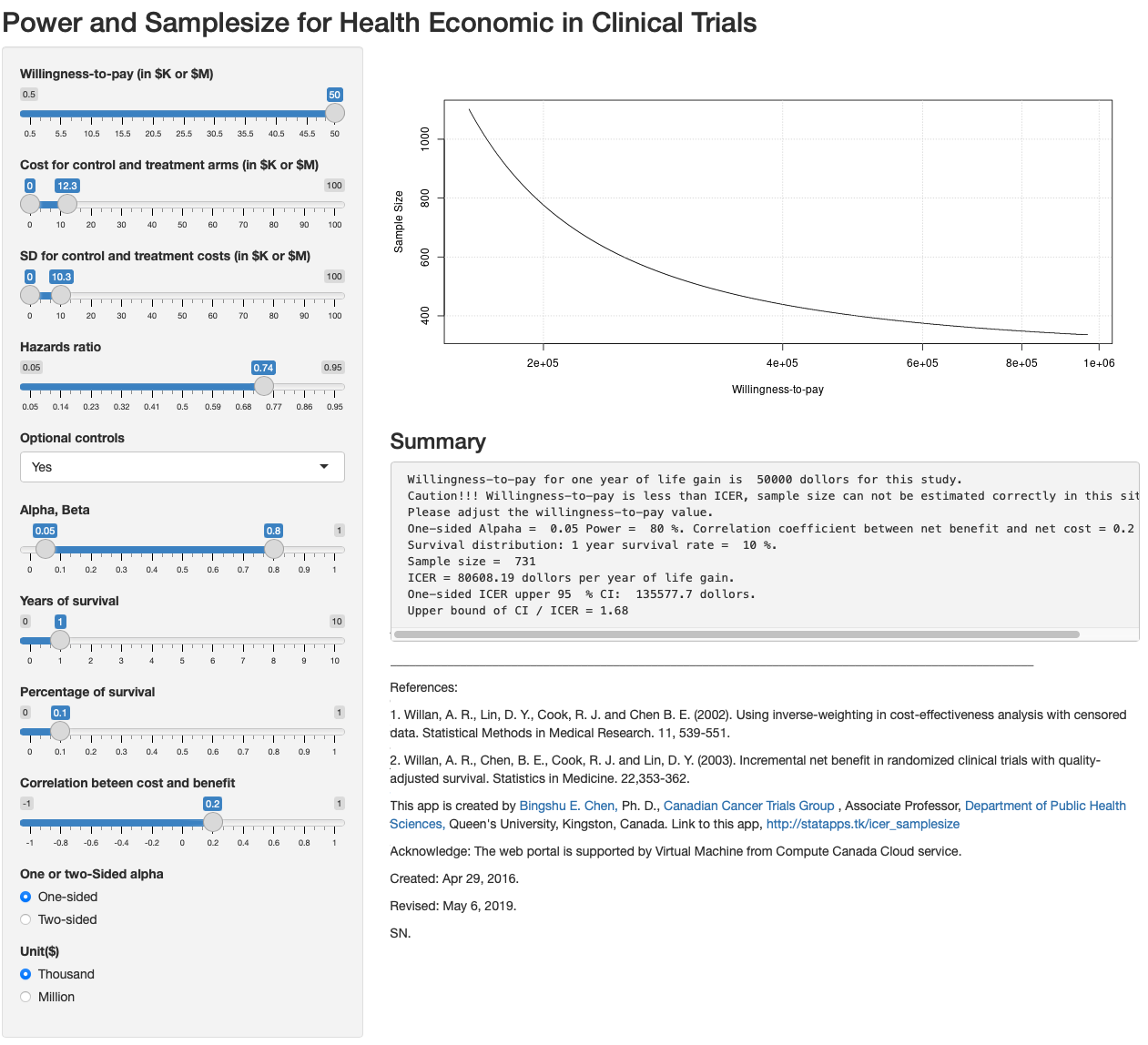


Legend: SD: standard deviation, ICER: incremental cost effectiveness ratio, CI: confidence interval,
